# Trends in tap and bottled water consumption among children and adults in the United States: analyses of NHANES 2011–16 data

**DOI:** 10.1186/s12937-020-0523-6

**Published:** 2020-01-29

**Authors:** Florent Vieux, Matthieu Maillot, Colin D. Rehm, Pamela Barrios, Adam Drewnowski

**Affiliations:** 1 MS-Nutrition, 27 bld Jean Moulin Faculté de Médecine la Timone, Laboratoire C2VN, 13385 Marseille, cedex 5 France; 2 Albert Einstein College of Medicine, Montefiore Medical Center, New York, NY 10467 USA; 3 Pepsico Inc, Purchase, New York, NY 10577 USA; 4 Center for Public Health Nutrition, University of Washington, Box 353410, Seattle, WA 98195 USA

**Keywords:** Water tap, Water bottled, Sugar-sweetened beverages, NHANES 2011–2016, Beverages, Time trends

## Abstract

**Background:**

Dietary Guidelines for Americans 2015–20 recommend choosing water in place of sugar-sweetened beverages (SSB). This study examined water consumption patterns and trends among children and adults in the US.

**Methods:**

Dietary intake data for 7453 children (4-18y) and 15,263 adults (>19y) came from two 24 h dietary recalls in three cycles of the National Health and Nutrition Examination Survey (NHANES 2011–2016). Water was categorized as tap or bottled (plain). Other beverages were assigned to 15 categories. Water and other beverage intakes (in mL/d) were analyzed by sociodemographic variables and sourcing location. Consumption time trends from 2011 to 2016 were also examined. Total water intakes from water, other beverages and moisture from foods (mL/d) were compared to Dietary Reference Intakes (DRI) for water.

**Results:**

Total dietary water (2718 mL/d) came from water (1066 mL/d), other beverages (1036 mL/d) and from food moisture (618 mL/d). Whereas total water intakes remained stable, a significant decline in SSB from 2011 to 2016 was fully offset by an increase in the consumption of plain water. The main sources of water were tap at home (288 mL/d), tap away from home (301 mL/d), and bottled water from stores (339 mL/d). Water and other beverage consumption patterns varied with age, incomes and race/ethnicity. Higher tap water consumption was associated with higher incomes, but bottled water was not. Non-Hispanic whites consumed most tap water (781 mL/d) whereas Mexican Americans consumed most bottled water (605 mL/d). Only about 40% of the NHANES sample on average followed US recommendations for adequate water intakes.

**Conclusion:**

The present results suggest that while total water intakes among children and adults have stayed constant, drinking water, tap and bottled, has been replacing SSB in the US diet.

## Introduction

The Dietary Guidelines for Americans 2015–2020 have encouraged consumers to choose beverages with no added sugars, such as water, in place of sugar-sweetened beverages (SSB) [1]. Dietary intake surveys in the US have pointed to a continuing decline in SSB consumption [2, 3], especially among children, teenagers, and young adults [3–6]. However, SSB consumption rates are still relatively high among racial/ethnic minorities and younger age groups and continue to be associated with higher obesity risk [3, 4, 6].

Replacing SSB with plain drinking water has become central to health promotion strategies, including those led by US federal agencies [1]. The Healthy, Hunger-Free Kids Act of 2010 [7] requires schools participating in the National School Lunch Program [8] to make free water available to students during meal times. The federal standards also require schools in the School Breakfast Program to make drinking water available when breakfast is served in the cafeteria [9]. Schools are encouraged to ensure that water fountains are clean and properly maintained, provide access to water fountains, dispensers, and hydration stations throughout the school, and allow students to have water bottles in class or to go to the water fountain [10]. Drinking fountains in public spaces are also viewed as an opportunity for public health. Much of the federal emphasis has been on plain drinking water from the tap.

Compared to the extensive literature on SSB consumption [2], less is known about consumption patterns and trends for drinking water, tap and bottled, among US children and adults. Past studies [11] have estimated that 56% of plain drinking water in the US comes from the tap, whereas 44% is bottled. Industry sources suggest that sales of bottled water have increased since then and that the global market for bottled water has grown substantially [12]. Analyses of the most recent National Health and Nutrition Examination Survey (NHANES) data would provide insights into the current tap and bottled water consumption trends in the US [11, 13].

The present hypothesis was that the reported decline in SSB consumption may have been offset, in part or in full, by a compensatory increase in drinking water. Such a finding would provide insight into the effectiveness of public health policies in the US [1]. The position of the Centers for Disease Control is that adequate water intakes would help increase overall water consumption, maintain hydration status, and reduce added sugar content of the diet, if substituted for SSB [10]. When it comes to maintaining hydration, examining compliance with standards and norms expressed either as water intakes in mL/d or as ratio on water intakes to calories (kcal/d) would be of additional interest. The question was whether any population subgroup was failing to maintain adequate water intakes, as specified by the Institute of Medicine (IOM).

The present analyses were based on the nationally representative NHANES 2011–2016 dietary intakes database for children (4-18y) and adults (≥19y) in the US, including the most recent 2015–2016 cycle [14]. The present goals were to examine consumption patterns for water and other beverages by socio-demographic variables, including education and incomes and to explore consumption trends between 2011 and 2016. The adequacy of total water intakes, as compared to the IOM recommendations was also examined.

## Methods

### Dietary intake databases

Consumption data for drinking water, beverages, and foods came from three cycles of the nationally representative NHANES, corresponding to years 2011–12, 2013–2014, and 2015–2016 [15]. The three NHANES cycles provided a nationally representative sample of 7453 children (aged 4-18y) and 15,263 adults (aged >19y).

The NHANES 24-h recall uses a multi-pass method, where respondents reported the types and amounts of all food and beverages consumed in the preceding 24-h from midnight to midnight [16]. The multi-pass method was conducted by a trained interviewer using a computerized interface [17]. Respondents first identified a quick list of foods and beverages consumed. The time and occasion for each food item was also obtained. A more detailed cycle then recorded the amounts consumed, followed by a final probe for any often-forgotten foods (beverages, condiments). Day one interviews were conducted by trained dietary interviewers in a mobile examination center. Day two interviews were conducted by telephone some days later [18].

For children 4-5y, dietary recall was completed entirely by a proxy respondent (i.e. parent or guardian with knowledge of the child’s diet) [16]. Proxy assisted interviews were conducted with children 6–11 years of age. Children 12-19y were the primary source of dietary recall but could be assisted by an adult who had knowledge of their diet.

We used a combination of the one-day value and the two-day mean to make use of all available dietary data. About 90% of people had two recalls. This method included all NHANES participants, even those without a second recall. Water consumers were defined as those NHANES participants who were drinking water on day one, day two, or both.

### Participant characteristics

NHANES participants were stratified by gender and age. The age group cut-points were: 4-8y, 9-13y, 14-18y, 19-30y, 31-50y, 51-70y, and > 70y. These age groups generally correspond to the age groups used by the IOM. Race/ethnicity was defined as: non-Hispanic white, non-Hispanic black, Mexican American, other Hispanic, and other/mixed race. Family income-to-poverty ratio (IPR) is the ratio of family income to the federal poverty threshold; the cut-points for IPR were < 1, 1–1.99, 2–3.49, and ≥ 3.5.

### Water intakes from water and other beverages

Water and other beverages were classified into groups. Plain drinking water was split into tap and bottled. Other beverages were classified as follows: milk and milk beverages, milk substitutes (soy milk), citrus juices, non-citrus juices, diet soda, regular soda, ready-to-drink tea, ready-to-drink coffee, fruit drinks, sports drinks, energy drinks, hot tea/coffee, alcoholic beverage, enhanced water, and supplemental beverages. Common examples by beverage category are presented in Table 5 in Appendix. The present analyses were for water from water and other beverages only. For example, milk consumed with cereal (i.e. not as a beverage) was assigned to the foods category.

The NHANES 24-h recalls for each participant provided information on the amount in grams of each food and beverage consumed [14]. The present results were for mL of water content from water and selected beverages and not for the volume of the beverages themselves (which may not be 100% water). Moisture from foods was calculated as well.

The USDA Food and Nutrient Database for Dietary Studies (FNDDS) was used to calculate the energy content of the diet based on caloric beverages and solid foods [19]. These data were used to calculate the ratios of water intakes (mL/d) to energy intakes (kcal/d) for each NHANES participant.

### Time trend analyses 2011–2016

Time trend analyses examined mean daily water intakes (mL/d) from tap and bottled water and water intakes from different beverage categories. Percent reported consumers for tap and bottled water was calculated as well, separately for each NHANES cycle.

### Determination of adequate water intakes

In conventional analyses, the mean of two 24-h recalls does not represent the habitual intakes of an individual. Failing to meet water or nutrient recommendations on a particular day may not accurately reflect the long-term status of the individual. For that reason, the National Cancer Institute (NCI) method was used to characterize the usual intake of water from water, beverages and foods [20–22]. This method has been used in other studies to estimate the usual intake of nutrients and food groups, including the population distribution of intakes.

### Sourcing location for water and other beverages

The following NHANES sourcing options for foods and other beverages were selected: store, work/school, someone else (e.g. friends’ home), quick-service restaurant (fast food), full-service restaurant, and other. Tap water does not have a source in NHANES; however, the source of water can be determined using information on the location of meals. For example, if a meal at a fast food restaurant was accompanied by tap water consumed at the same time, we can infer that the tap water was from “away from home”. Tap water consumed at home was coded as “at home”. For bottled water we created a variable called “home” or combined “store and home”, as most home-consumed foods are from the store. Tap water category “outside of home” was reserved for occasions when tap water was consumed away from home and with no other foods at the same time.

### Data availability and ethical approval

The necessary IRB approval for NHANES had been obtained by the National Center for Health Statistics (NCHS) [23]. Adult participants provided written informed consent. Parental/ guardian written informed consent was obtained for children. Children/adolescents ≥12y provided additional written consent. All NHANES data are publicly available on the NCHS and USDA websites [14]. Per University of Washington (UW) policies, public data do not involve “human subjects” and their use requires neither IRB review nor an exempt determination. Such data may be used without any involvement of the Human Subjects Division or the UW Institutional Review Board.

### Statistical analyses

The survey-weighted mean intakes of total water were evaluated overall and by age group, gender, race/ethnicity, and family income-to-poverty ratio. All analyses accounted for the complex survey design of NHANES and reflect dietary behaviors of the US adult population from 2011 to 16.

Analyses of what proportion of NHANES 2011–2016 participants met the IOM recommendations for adequate hydration were based on habitual water intakes established using the National Cancer Institute method [24, 25]. The probability of water consumption was estimated to be 1. Subsequent analyses specified the consumption-day amount using linear regression and intake data from 24-h recalls.

The consumption of tap and bottled water was evaluated separately for the entire population and for population sub-groups. Survey-weighted means and corresponding standard errors were obtained. Hypothesis testing was based on a linear trend test which treats the NHANES cycles as a continuous variable. This trend test may be sensitive to extreme values in either the first (2011–12) or last (2015–2016) cycle and may not reflect weak non-linear trends. All analyses were conducted using SAS software, version 9.4 (SAS Institute Inc., Cary NC, USA) by using SURVEYREG, SURVEYMEANS and SURVEYFREQ procedures.

## Results

### Total water intakes from water, other beverages, and foods

Table 1 shows total water intakes in mL/d by gender, age, socio-demographic groups, and eating occasion. Data are for total water intakes, water from water and other beverages, and moisture from foods. Total water intake was 2718 mL/d, of which 2100 mL/d (77%) came from water and other beverages and 618 mL/d (23%) came from food moisture. Men had higher water intakes from all sources than did women (2949 mL vs. 2495 mL; *p* < 0.01). There was also a strong age effect. Total water intakes increased sharply with age, peaked for the 31–50y age group, and declined thereafter, dropping to 2355 mL/d after the age of 70y.

**Table 1:** Water intakes (mL/d) from water, other beverages and foods (mean, standard error) by individual characteristics and meal occasion

	Water, other beverages, and foods (mL/d)	Water and other beverages (mL/d)	Food moisture (mL/d)
All >4y (*N = 22,716*)	2718 (27)	2100 (26)	618 (6)
Gender			
Males (*N* = 11,206)	2949 (36)	2285 (34)	664 (6)
Females (*N* = 11,510)	2495 (25)	1921 (24)	574 (7)
*p*-value for effect	< 0.01	< 0.01	< 0.01
Age			
4-8y (*N* = 2644)	1441 (18)	938 (16)	503 (8)
9-13y (*N* = 2501)	1691 (23)	1143 (20)	547 (8)
14-18y (*N* = 2308)	2128 (47)	1609 (44)	518 (8)
19-30y (*N* = 3248)	2936 (52)	2331 (48)	606 (10)
31-50y (*N* = 5071)	3166 (36)	2513 (35)	652 (9)
51-70y (*N* = 4873)	2997 (43)	2339 (42)	658 (7)
> 70y (*N* = 2071)	2355 (28)	1707 (27)	648 (10)
*p*-value for effect	< 0.01	< 0.01	< 0.01
*p*-trend	< 0.01	< 0.01	< 0.01
Race/ethnicity			
non-Hispanic white (*N* = 7802)	2879 (31)	2266 (29)	613 (7)
non-Hispanic black (*N* = 5365)	2249 (36)	1695 (32)	554 (7)
Mexican American (*N* = 3698)	2487 (51)	1857 (48)	630 (10)
Other Hispanic (*N* = 2473)	2505 (38)	1897 (34)	609 (8)
Other/mixed race (*N* = 3378)	2635 (38)	1899 (38)	737 (13)
*p*-value for effect	< 0.01	< 0.01	< 0.01
IPR			
< 1 (*N* = 5633)	2461 (49)	1888 (48)	573 (6)
1–1.99 (*N* = 5545)	2579 (38)	1989 (36)	590 (8)
2–3.49 (*N* = 4209)	2692 (40)	2092 (39)	601 (9)
> 3.49 (*N* = 5558)	2952 (38)	2288 (37)	664 (9)
Missing (*N* = 1771)	2616 (56)	1989 (52)	627 (13)
*p*-value for effect	< 0.01	< 0.01	< 0.01
*p*-trend	< 0.01	< 0.01	< 0.01

Non-Hispanic whites had highest total water intakes (2879 mL/d) and highest water intakes from water and other beverages (2266 mL/d). Non-Hispanic blacks had lowest total water intakes (2249 mL/d) and lowest water intakes from water and other beverages (1695 mL/d). Total water intakes followed a socioeconomic gradient. Groups with higher IPR had the highest total water intakes (2952 mL/d), had highest intakes of water and other beverages (2288 mL/d) and derived most moisture from foods (664 mL/d). The difference in total water intakes between IPR < 1 (2461 mL/d) and IPR > 3.49 was almost 500 mL/day. Adjusting for age, intakes were 2225 mL/d in the lowest IPR and 2548 mL/d in the highest IPR.

### Water intakes from water and other beverages

Table 2 shows intakes of water, now separated into tap and bottled water, by individual socio-demographic variables and eating occasion. Total intake of drinking water, both tap and bottled, was 1066 mL/d. Tap water supplied 661 mL/d whereas bottled water supplied 404 mL/d. Other beverages, caloric and non-caloric, supplied another 1034 mL/d. Thus, the daily distribution of dietary sources of water was tap water (24%), bottled water (14%), other beverages (38%), and food moisture (23%).

**Table 2:** Water intakes (mL/d) from water and other beverages (mean, standard error) by socio-demographics and sourcing location

	Water (tap+bottled)	Tap water	Bottled water	Other beverages
All >4y (*N* = 22,716)	1066 (20)	661 (24)	404 (16)	1034 (17)
Gender				
Males (*N* = 11,206)	1071 (24)	676 (25)	395 (16)	1214 (24)
Females (*N* = 11,510)	1060 (20)	647 (27)	414 (17)	861 (14)
*p*-value for effect	0.59	0.15	0.10	< 0.01
Age				
4-8y (*N* = 2644)	430 (15)	264 (18)	165 (12)	508 (11)
9-13y (*N* = 2501)	577 (19)	359 (21)	218 (18)	567 (12)
14-18y (*N* = 2308)	866 (34)	481 (34)	385 (21)	743 (24)
19-30y (*N* = 3248)	1305 (44)	819 (47)	486 (24)	1026 (21)
31-50y (*N* = 5071)	1297 (30)	772 (33)	525 (25)	1217 (28)
51-70y (*N* = 4873)	1100 (28)	694 (32)	406 (20)	1239 (22)
> 70y (*N* = 2071)	809 (24)	598 (29)	212 (14)	898 (20)
*p*-value for effect	< 0.01	< 0.01	< 0.01	< 0.01
*p*-trend	< 0.01	< 0.01	< 0.01	< 0.01
Race/ethnicity				
non-Hispanic white (*N* = 7802)	1109 (26)	781 (27)	328 (16)	1157 (23)
non-Hispanic black (*N* = 5365)	894 (29)	359 (23)	534 (23)	802 (14)
MexicanAmerican (*N* = 3698)	997 (36)	391 (21)	605 (26)	861 (19)
Other Hispanic (*N* = 2473)	1041 (33)	497 (41)	544 (34)	856 (18)
Other/mixed race (*N* = 3378)	1092 (26)	658 (31)	434 (29)	807 (24)
*p*-value for effect	< 0.01	< 0.01	< 0.01	< 0.01
Income to poverty ratio (IPR)				
< 1 (*N* = 5633)	932 (37)	496 (45)	436 (21)	956 (30)
1–1.99 (*N* = 5545)	1003 (27)	555 (27)	448 (22)	986 (27)
2–3.49 (*N* = 4209)	1036 (28)	643 (28)	394 (21)	1055 (25)
> 3.49 (*N* = 5558)	1181 (29)	821 (32)	360 (20)	1108 (19)
Missing (*N* = 1771)	1074 (49)	609 (44)	464 (29)	916 (27)
*p*-value for effect	< 0.01	< 0.01	< 0.01	< 0.01
*p*-trend	< 0.01	< 0.01	< 0.01	< 0.01

Men and women drank comparable amounts of tap and bottled water. There was a significant effect of age group. The consumption of tap and bottled water peaked at ages 31-50y and declined thereafter. People > 70 y old drank less water than other groups; there was an age-related decline in bottled water (212 mL/d) and other beverages (898 mL/d).

Although the consumption of drinking water increased with rising IPR, the income effect operated in opposite directions for tap water and for bottled water. Higher IPR was associated with more tap water (from 495 to 821 mL/d) but, unexpectedly, with somewhat lower consumption of bottled water (from 436 to 360 mL/d).

There were also differences in consumption of drinking water by race/ethnicity. Non-Hispanic whites consumed the most tap water and the least bottled water (781 mL/d and 328 mL/d, respectively). Mexican Americans and non-Hispanic blacks consumed more bottled water than tap water, as did other Hispanics.

Men consumed more other beverages than did women (1214 mL/d vs. 861 mL/d). Beverage consumption peaked at 51-70y and declined for >70y age group. Non-Hispanic whites drank the highest amounts of other beverages (1157 mL/d); non-Hispanic blacks drank the least (803 mL/d). Water consumption from other beverages increased with IPR.

Separate analyses of caloric SSB, estimated water consumption from SSB at 288 mL/d, with peak consumption observed among younger adults aged 19-30y (410 mL/d). Non-Hispanic blacks consumed the most water from SSB (368 mL/d); the other/mixed race group consumed the least (205 mL/d). Water from SSB was lower among higher income groups (209 mL/d) as compared to 390 mL/d among lowest income groups.

### Time trends in SSB and water consumption 2011–2016

Table 3 shows time trends for water intake from water and other beverages for each NHANES cycle (2011–2016) and separately for children (4-18y) and for adults (>19y). Total water intakes did not change during this time period, remaining at approximately 2100 mL/d. However, there was a significant decline in the consumption of other beverages (from 1097 mL/d to 970 mL/d) that was especially pronounced for children (from 675 mL/d to 522 mL/d) and was significant for both children and for adults. That reduction in intake was largely caused by reduced consumption of SSB, which was highly significant among both children and adults. Children also consumed less water from non-SSB beverages. Analyses for trends were significant.

**Table 3:** Time trends in water intakes (mL) from tap, bottled water, and other beverages including SSV (mean, standard error)

	NHANES cycle				*p*-trend
	2011–12	2013–14	2015–16	*p*-value	
All >4y					
Tap+Bottled water	1011 (33)	1039 (30)	1144 (38)	<0.05	<0.05
Tap water	643 (32)	639 (48)	700 (43)	0.51	0.29
Bottled water	368 (30)	400 (27)	444 (24)	0.14	0.05
Other beverages	1097 (31)	1038 (32)	970 (21)	<0.01	<0.005
SSB	322 (12)	283 (14)	262 (13)	<0.01	<0.005
Not SSB	775 (32)	755 (31)	708 (21)	0.17	0.09
Total	2108 (45)	2077 (44)	2114 (46)	0.82	0.92
Age 4-18y					
Tap+Bottled water	577 (29)	642 (24)	663 (34)	0.12	0.06
Tap water	332 (26)	383 (36)	393 (35)	0.30	0.17
Bottled water	245 (31)	259 (18)	269 (23)	0.82	0.53
Other beverages	675 (21)	630 (18)	522 (13)	<.0001	< 0.0001
SSB	324 (9)	291 (16)	237 (14)	<.0001	< 0.0001
Not SSB	351 (18)	339 (15)	285 (8)	<0.001	<0.005
Total	1253 (38)	1272 (30)	1185 (30)	0.11	0.16
Age 19y+					
Tap+Bottled water	1126 (38)	1143 (36)	1271 (40)	<0.05	<0.05
Tap water	725 (38)	706 (54)	781 (45)	0.50	0.35
Bottled water	400 (31)	437 (30)	490 (28)	0.11	<0.05
Other beverages	1208 (35)	1144 (35)	1088 (27)	<0.05	<0.01
SSB	321 (14)	280 (15)	269 (14)	<0.05	<0.05
Not SSB	887 (37)	864 (34)	819 (25)	0.27	0.13
Total	2334 (49)	2287 (50)	2359 (51)	0.60	0.73

While total water intakes remained stable, the significant decline in SSB consumption was offset by a corresponding significant increase in the consumption of tap and bottled water. Among adults aged >19y, the increase in tap and bottled water consumption from 1126 mL/d to 1271 mL/d was statistically significant. A smaller increase (from 577 mL/d to 663 mL/d) among children aged 4-18y was not. The observed small increases in bottled water did not reach statistical significance.

**Fig. 1:**
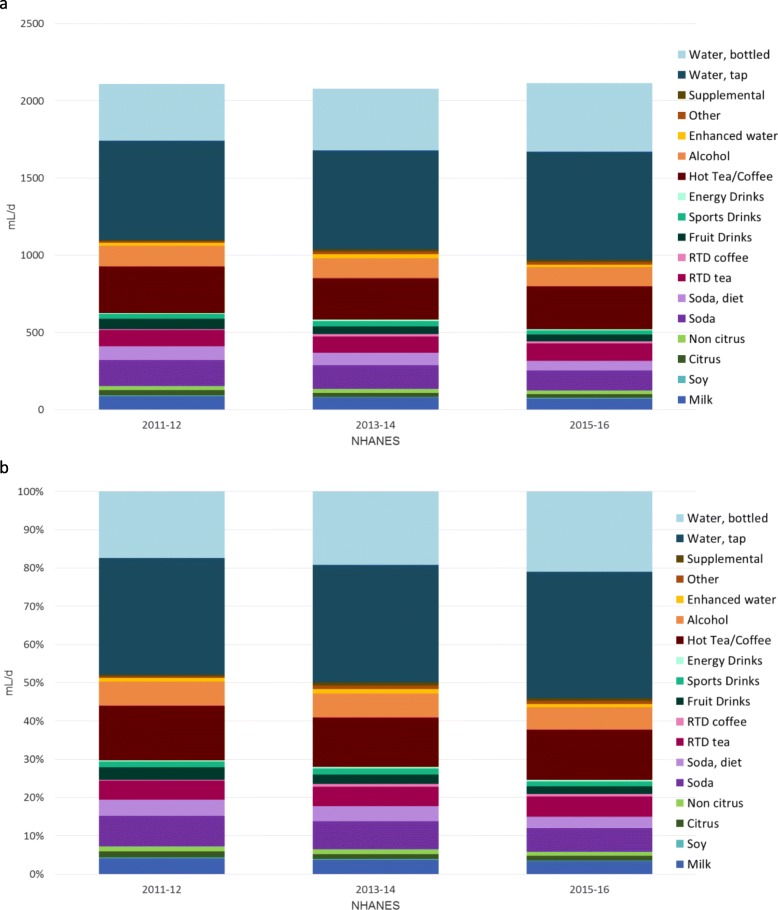
**ab**. (**a**) Absolute intakes in mL/d of water (tap and bottled) and other beverages and (**b**) percent contribution from water (tap and bottled) and other beverages by NHANES year (2011–2016) and beverage category. Data are for children and adults aged >4y

Figure 1 shows time trends for water intakes from water and other beverages for each of the three NHANES cycles. Water has been separated into tap and bottled, whereas beverages are shown by category. The SSB category includes regular sodas, fruit drinks and ready to drink tea and coffee, and most sports and energy drinks. The data show that a progressive decline in beverages, including SSB has been compensated for by drinking water. Figure 1a **(top)** shows absolute amounts in mL/d, whereas Fig. 1b **(bottom)** shows the percent contribution of each water source to water intakes.

### Water intakes from water and other beverages by age

**Fig. 2:**
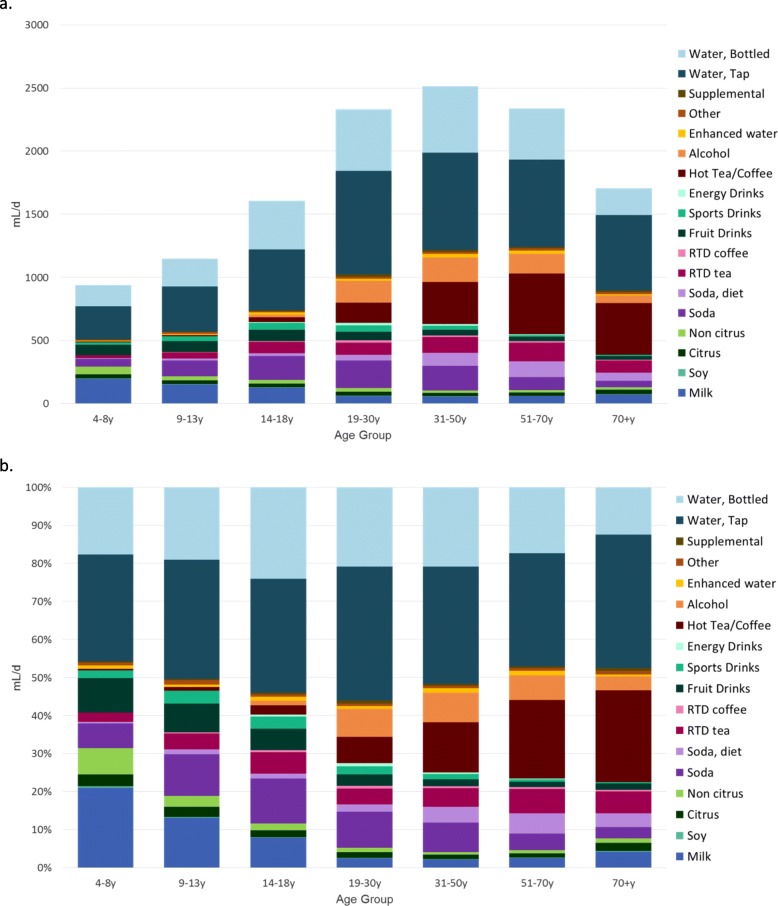
**ab**. (**a**) Absolute intakes in mL/d of water (tap and bottled) and other beverages and (**b**) percent contribution from water (tap and bottled) and other beverages by age group and beverage category

Figure 2 summarizes water intakes from tap and bottled water and from different other beverage categories by age. The categories are listed in the figure key. Figure 2a **(top)** shows water intakes in mL/d, whereas Fig. 2b **(bottom)** shows the percent contribution of different beverages to water intakes for each age group.

Water, tap and bottled, provided about 50% of water intakes, excluding moisture from food. Caloric and non-caloric beverages provided between 508 mL/d and 1239 mL/d of water depending on age. The type of beverages consumed varied as a function of age. Figure 2a shows that the consumption of milk was highest for the 4-8y age group and declined progressively with age. The consumption of 100% non-citrus juices (driven in large part by apple juice) and fruit drinks also declined with age. By contrast, the consumption of regular soda increased with age, peaked in early adult life (19-30y) and declined thereafter. The consumption of diet soda increased sharply after the age of 30y. The consumption of 100% citrus juice did not show much variation with age.

The consumption of presweetened ready-to-drink (RTD) tea increased with age. The consumption of RTD coffee was low. Sports drinks and energy drinks were consumed primarily by adolescents and by young adults. The consumption of brewed coffee and tea increased sharply after the age of 18y and so did the consumption of alcohol. Older adults (>70y) were getting as much as 412 mL/d of daily water (or 24% of water from beverages) from coffee and tea.

Figure 2b shows clearly the age-related trends in water and beverage consumption. First, the consumption of milk, 100% fruit juice, and juice drinks declined with age, whereas the consumption of SSB and water increased. The 19–30y age group derived most water from tap and bottled water but also from SSB. However, both tap and bottled water consumption declined with age, replaced by tea and coffee and, to a lesser extent, by alcohol.

**Fig. 3:**
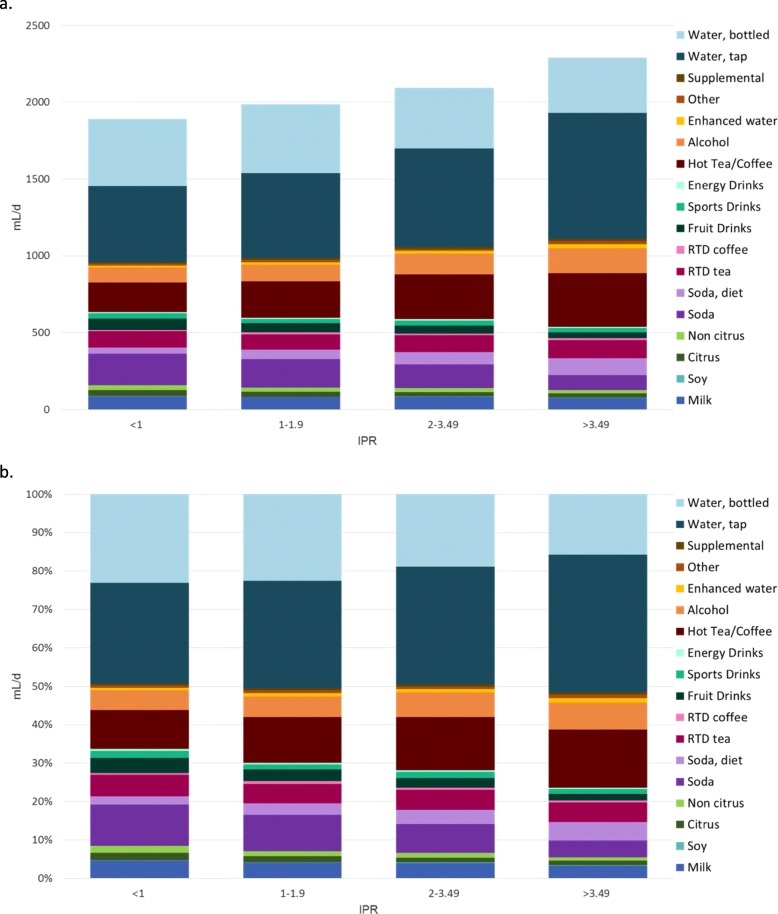
**ab**. (**a**) Absolute intakes in mL/d of water (tap and bottled) and other beverages and (**b**) percent contribution from water (tap and bottled) and other beverages by IPR and beverage category

These age-related trends were compounded by socioeconomic status. Figure 3 shows that the amounts and types of beverages consumed varied with IPR. Figure 3a **(top)** shows water intakes in mL/d, whereas Fig. 3b **(bottom)** shows the percent contribution of different beverages to water intakes for each IPR group. There was a small IPR linked decline in the consumption of milk and citrus and non-citrus juices. There was a sharp drop in the consumption of SSB that was partly offset by higher consumption of diet soda. The proportion of water from fruit drinks, sports drinks, and energy drinks declined. By contrast higher IPR was associated with higher percent contributions from brewed coffee and tea, enhanced water, and alcohol.

### Source locations of water and other beverages

**Fig. 4:**
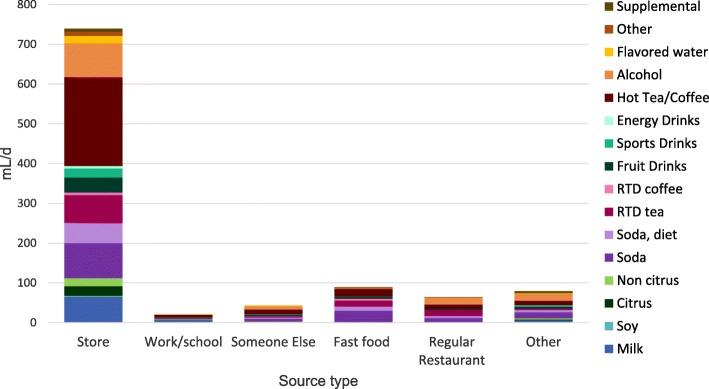
Percentage of participants meeting IOM adequate intake recommendations for water by age group

Most bottled water and other beverages (1076 mL/d or 75% of total) came from the store. Of this, 339 mL/d came from store-bought bottled water and 737 mL/d came from store bought other beverages. Figure 4 shows the distribution of store bought other beverages by category. Smaller amounts of other beverages and virtually no bottled water were obtained from restaurants (full-service [FSR] and quick-service [QSR]), work/school or someone else.

Tap water consumption (589 mL/d) was almost evenly split between tap water at home (288 mL/d) and tap water away from home (301 mL/d); as previously noted, it was not possible to differentiate tap water more finely due to the manner of data collection.

### Meeting recommendations for adequate water intakes

**Fig. 5:**
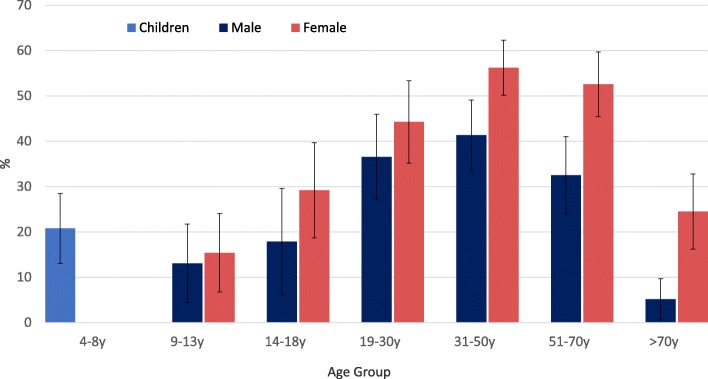
Distribution of other beverages by sourcing location and beverage category. NHANES 2011–2016

These data analyses used the NCI method to establish habitual water intakes based on two 24 h recalls, following published procedures [25]. Based on the NCI method, only about 40% of the NHANES 2011–2016 sample met the IOM recommendations for adequate water intakes. Figure 5 shows the percent of participants within each age group that met IOM recommendations for adequate water intakes using the NCI method. Data for years 2011–2016 were pooled as there was little year-to-year variation. The data show that men aged >70y were least likely to meet the IOM recommendations; only 5.15% did so.

Table 4 shows that the observed water volume per 1000 kcal for most groups was between 1.4–1.6 L/1000 kcal, consistent with the desirable values (≥1.0 L/1000 kcal) as recommended by the IOM and by the European Food Safety Authority [24–26]. The only two age groups with values < 1.0 L/1000 kcal were children aged 4–13y. Higher water volume per 100 kcal was observed for non-Hispanic Whites and for groups with higher IPR. Non-Hispanic Black participants and lower IPR groups had lowest water volumes per 1000 kcal.

**Table 4:** Water density (mL/1000 kcal) by gender, age, race/ethnicity, and IPR

	Water density (mL/1000 kcal)
All participants >4y (*N* = 22,716)	
Gender	
Males (*N* = 11,206)	1333 (14)
Females (*N* = 11,510)	1551 (16)
test	< 0.59
Age	
4-8y (*N* = 2644)	854 (9)
9-13y (*N* = 2501)	916 (12)
14-18y (*N* = 2308)	1151 (27)
19-30y (*N* = 3248)	1457 (27)
31-50y (*N* = 5071)	1609 (20)
51-70y (*N* = 4873)	1640 (23)
> 70y (*N* = 2071)	1445 (21)
test	< 0.01
Race/ethnicity	
non-Hispanic white (*N* = 7802)	1517 (16)
non-Hispanic black (*N* = 5365)	1239 (27)
Mexican American (*N* = 3698)	1276 (26)
Other Hispanic (*N* = 2473)	1388 (27)
Other/mixed race (*N* = 3378)	1445 (20)
test	< 0.01
IPR (cutpoints TBD)	
< 1 (*N* = 5633)	1348 (24)
1–1.99(*N* = 5545)	1398 (21)
2–3.49 (*N* = 4209)	1433 (27)
> 3.49 (*N* = 5558)	1513 (18)
Missing (*N* = 1771)	1497 (40)
test	< 0.01

## Discussion

The present analyses, based on the most recent 2015–2016 NHANES data confirm that the consumption of SSB in the US continues to drop [1, 6, 27, 28]. While SSB may not be the biggest source of dietary energy for anyone except teenagers, they are the main source of added sugars in the American diet [29]. The Dietary Guidelines for Americans 2015–20, along with a number of industry-led initiatives specifically called for making water the beverage of choice [1, 30]. Replacing caloric SSB with plain drinking water has become a priority area for interventions in school and public health nutrition [6]. Sales of bottled water in the US are also reported to be on the rise [31].

The present analyses provide several new insights into water and other beverage consumption patterns and trends. First, total dietary water intakes from drinking water, other beverages, and foods have remained stable from 2011 to 2016. Total amounts of water and other beverages have also remained constant. We now show for the first time that the observed significant drop in SSB consumption has been offset by an increased consumption of plain drinking water. On the average, about 62% of drinking water came from the tap, a major increase from 56% observed in the 2005–2010 NHANES database [11].

Second, and contrary to expectations, tap water consumption was higher at higher incomes, whereas the consumption of bottled water was higher at lower incomes. Consistent with previous findings, non-Hispanic whites and higher income groups in the present study consumed the largest amounts of tap water [11]. By contrast, Mexican Americans drank the most bottled water and the least tap water. In previous analyses of NHANES data, water consumption, both bottled and tap, among adults was significantly associated with higher education and incomes. The present data showed that the income effect (IPR) now operated in opposite directions.

The continuing positive social gradient in tap water consumption is a cause for concern. Whereas bottled water is purified or filtered, packaged, sealed, and distributed through retail channels, municipal tap water is delivered through pipes from reservoirs, rivers or aquifers. The quality of tap water has been variable and found to be problematic, especially in lower-income areas [32, 33]. There seems to be a growing perception, confirmed by research studies, that tap water is safe to drink only in affluent neighborhoods [32, 34]. The CDC does not advise tap water for anyone with a compromised immune system [35]. Additional studies have pointed to concerns with copper and lead [36]. Making water the beverage of choice needs to be sensitive to the quality of the local water supply and to community resources, wants, and needs.

The inverse social gradient in bottled water consumption seems to be unique to the US. Studies with children in the UK showed that bottled water consumption was associated with higher household socioeconomic status (SES) [37, 38]. A social gradient for bottled water was not observed in France [39]. A previous study based on French INCA data examined water and other beverage consumption patterns by breakfast, AM snack, lunch, PM snack, dinner, and evening snack [39]. Another study based on data from the UK used time intervals to determine temporal distribution of water and beverage intakes [37]. There is a need for more international comparisons on who drinks tap water as opposed to bottled water [40], with what meal, and at what time of day [11, 37, 39].

The present analyses further showed that the consumption of bottled water was strongly age dependent [11, 13]. Highly consumed by teenagers and young adults, bottled water was replaced in later life by tea and coffee and to a lesser extent alcohol. Older adults > 70 y drank relatively little bottled water.

Finally, bottled water came mostly from the store. Very little bottled water came from fast food or full-service restaurants, or from work or school. This may change as schools may begin to use bottled water due to safety concerns [34, 36, 40]. By contrast, tap water was evenly split between tap at home and tap away from home.

The present analyses based on the NCI method showed that the IOM recommendations for adequate water intakes (AI) were met by only 40% of the US population on average, with numbers varying from 5 to 50% depending on age. The IOM AI values are set at 1700 mL/d for boys and girls in the 4-8y age group and 2.1 L/d for girls and 2.4 L/d for boys in the 9-13y age group [24]. For 14–18 year-olds, the AI values are 3.3 L/d for boys and 2.3 L/d for girls. The IOM reference values for water intake among adults are 2.7 L/d for women and 3.7 L/d for men [24]. The IOM AI goals are derived from the median intake of the US population whereas the European Food Safety Authority (EFSA) recommends a daily total water intake (water from food and beverages) of 2.5 L for men and 2.0 L for women to maintain urinary osmolality of 500 mOsmol/L.

In past analyses of NHANES 2005–2010 [11], younger adults exceeded or came close to satisfying the DRIs for water. The shortfall between IOM recommendations and reality has been reported as most acute for young children and for older adults [11, 13]. Older men and women failed to meet the Institute of Medicine (IOM) AI values, with a shortfall in daily water intakes of 1218 mL and 603 mL respectively. Eighty-three percent of women and 95% of men ≥71y failed to meet the IOM AI values for water [11].

However, it must be noted that non-compliance with IOM guidelines does not indicate dehydration. The second criterion of adequate hydration, water volume in mL per 1000 kcal, did not fall short of desirable values. Whereas the standard IOM recommendation is at least 1.0 L per 1000 kcal, the observed values of ~ 1,5 L/1000 kcal were well above this threshold [24]. In NHANES 2005–2010 also, average water volume per 1000 kcal was 1.2–1.4 L/1000 kcal for most population sub-groups, higher than the suggested minimum levels of 1.0 L/1.000 kcal [11].

On the other hand, beverage consumption in the US has shown a steady decline. The decline has been most acute for SSB but has also been observed with milk and 100% orange juice. While the replacement of SSB with plain water does have the benefit of removing added sugar, consistent with the Dietary Guidelines goals, the replacement of milk or 100% juice with water may not have the same nutritional benefits. Total water intake below IOM recommended levels may also be a cause for concern, especially among older adults.

The present analyses had limitations. First, the NHANES data are based on self-report and are subject to random and systematic reporting errors. In particular, a 24-h recall may systematically underestimate water and other beverage intake, especially outside of meals since it is very difficult for individuals to remember exactly how much tap water they had outside of meals. Fluid-specific records, used in smaller scale studies, may provide higher quality data. The use of proxy respondents for children ages 4-5y and proxy assisted interviews for children 6–11 make the collection of accurate data especially challenging. The 2 days of dietary recalls used different methods to collect the data, which may affect the estimates of water consumption. Underreporting of water intakes would lead to overestimating the percent of adults who fail to meet the recommended intakes. However, the NHANES has the advantage of being based on a large, nationally representative population sample. The NHANES dataset forms the basis for dietary surveillance in the US.

## Conclusion

The SSB in the US diet are in the process of being replaced by plain water, both tap and bottled. This is an encouraging finding when it comes to public health goals. However, water consumption patterns showed wide variations by socioeconomic status, age group, and race/ethnicity. Social marketing strategies to promote water consumption, tap or bottled, will need to take these factors into account.

## Data Availability

Data used in the study is publicly available through the NHANES database (at https://wwwn.cdc.gov/nchs/nhanes/continuousnhanes/default.aspx).

## Appendix

**Table 5:** Common examples by beverage category

Beverage category	Most commonly reported examples
Water, tap	Tap water
Water, bottled	Bottled water (still)
Milk and milk beverages	Whole milk, 2% milk, 1% milk
Milk substitutes	Soy milk, almond milk
Citrus juices	Orange juice (canned/bottled), orange juice (w/calcium), orange juice (NFS^a^)
Non-citrus juices	Apple juice, grape juice, pineapple juice, fruit juice blend, fruit juice (NFS^a^)
Soda, regular	Sugar-sweetened beverages, carbonated
Soda, diet	Sweetened beverages, carbonated
Ready to drink (RTD) tea	pre-sweetened tea, tea NFS^a^ presweetened with sugar
Ready to drink (RTD) coffee	pre-sweetened coffee, coffee NFS^a^ presweetened with sugar
Fruit drinks (regular & diet)	Fruit juice drink, fruit flavored drink (made from powder), fruit juice drink (w/vitamin C)
Sports drinks (regular & diet)	Gatorade, Powerade
Energy drinks (regular & diet)	Red Bull
Coffee and tea	Regular coffee (from ground), decaffeinated coffee (from instant), unsweetened tea
Flavored & enhanced water	Carbonated water (unsweetened), Glaceau water, sweetened carbonated water (e.g., tonic)
Alcoholic beverages	Beer, lite beer, red wine
Other beverages	Meal replacement beverages

^a^ *NFS* Not further specified

## Abbreviations

FNDDS: USDA Food and Nutrient Database for Dietary Studies
FSR: Full-service restaurant
IOM: Institute of Medicine
IPR: Income-to-Poverty Ratio
NCI: National Cancer Institute
NHANES: National Health and Nutrition Examination Survey
QSR: Quick-service restaurant
RTD: Ready-to-drink
SES: Socioeconomic status
SSB: Sugar-Sweetened Beverages
